# PRDX2 promotes the proliferation of colorectal cancer cells by increasing the ubiquitinated degradation of p53

**DOI:** 10.1038/s41419-021-03888-1

**Published:** 2021-06-11

**Authors:** Wuyi Wang, Jinlai Wei, Hairong Zhang, Xiangru Zheng, He Zhou, Yajun Luo, Jianguo Yang, Qican Deng, Siqi Huang, Zhongxue Fu

**Affiliations:** Department of Gastrointestinal Surgery, First Affiliated Hospital of Chongqing Medical University, Chongqing, China

**Keywords:** Colorectal cancer, Colorectal cancer

## Abstract

Colorectal cancer is the most common gastrointestinal cancer and causes severe damage to human health. PRDX2 is a member of the peroxiredoxin family reported to have a high level of expression in colorectal cancer. However, the mechanisms by which PRDX2 promotes the proliferation of colorectal cancer are still unclear. Here, the results indicated that PRDX2 expression was upregulated in colorectal cancer and closely correlated with poor prognosis. Functionally, PRDX2 promoted the proliferation of colorectal cancer cells. Mechanistically, PRDX2 could bind RPL4, reducing the interaction between RPL4 and MDM2. These findings demonstrate that the oncogenic property of PRDX2 may be attributed to its regulation of the RPL4-MDM2-p53 pathway, leading to p53 ubiquitinated degradation.

## Introduction

In 2018, approximately 1.8 million new colorectal cancer patients were diagnosed globally, and the disease caused 881,000 deaths. Colorectal cancer has a high morbidity and mortality rate, ranking third and second, respectively, among all cancers [1]. Data show that 521,490 cases of colorectal cancer emerged in China in 2018, causing 247,563 deaths [2]. Although significant progress has been made in diagnosis and treatment, long-term survival rates are still unsatisfactory [3].

Reactive oxygen species (ROS) are the byproducts of cell metabolism [4, 5]. Elevated intercellular ROS levels can activate tumor-promoting signals, promote cell proliferation, enhance cell survival, and induce DNA damage [6]. Besides, ROS can also promote antitumor signals and induce tumor apoptosis [7]. Tumor cells usually express a high level of antioxidant proteins to maintain the redox balance. PRDX2 is a member of the peroxiredoxins (PRDXs) family that has been reported to have a high level of expression in many cancers, such as colorectal cancer, gastric cancer, and lung cancer [8–10]. Patients with high PRDX2 expression had a worse prognosis compared to those with low expression. However, the biological functions of PRDX2 in cancer have not been completely understood.

Posttranscriptional modification (PTM) is one of the mechanisms for adaption to rapid changes in the internal and external environments [11, 12]. The ubiquitin-proteasome system (UPS) is the primary system responsible for protein degradation. The UPS controls many different cellular processes by selectively degrading proteins and plays a crucial role in regulating a wide range of cytological and physiological processes [13, 14]. Ubiquitin-ligase enzymes function sequentially via activation (E1), conjugation (E2), and ligation (E3) to catalyze the covalent attachment of ubiquitin to lysine residues on substrates [15]. Ubiquitination is a precisely controlled process. Deubiquitinating enzymes not only inhibit the ubiquitination process but also recycle ubiquitin molecules and proofread ubiquitin conjugates [15]. Thus, together with the ubiquitination system, they form an intricate network affecting almost all cell biological processes.

p53 is a critical tumor suppressor. It is a central mediator that controls cell proliferation and apoptosis [16, 17]. Activation of the p53 gene leads to the expression of many tumor suppressor genes, while mutations or deletions of the p53 gene cause disturbances in intracellular signaling pathways, uncontrolled cell growth, and apoptosis [18]. MDM2 is an E3 ubiquitin-ligase enzyme and is the primary negative regulator of the p53 protein. RING at the carboxyl terminus of MDM2 catalyzes the ubiquitination of p53 and leads to its degradation by the 26S proteasome [19–21].

In this study, we first investigated the relationship between PRDX2 and p53 in colorectal cancer. Here, we report that PRDX2 is a novel regulator of the RPL4-MDM2-p53 loop. Our findings provide novel insights into the role of PRDX2 in promoting colorectal cancer development and suggest that PRDX2 could be a promising target for colorectal cancer treatment.

## Results

### PRDX2 is overexpressed in colorectal cancer

**Fig. 1:**
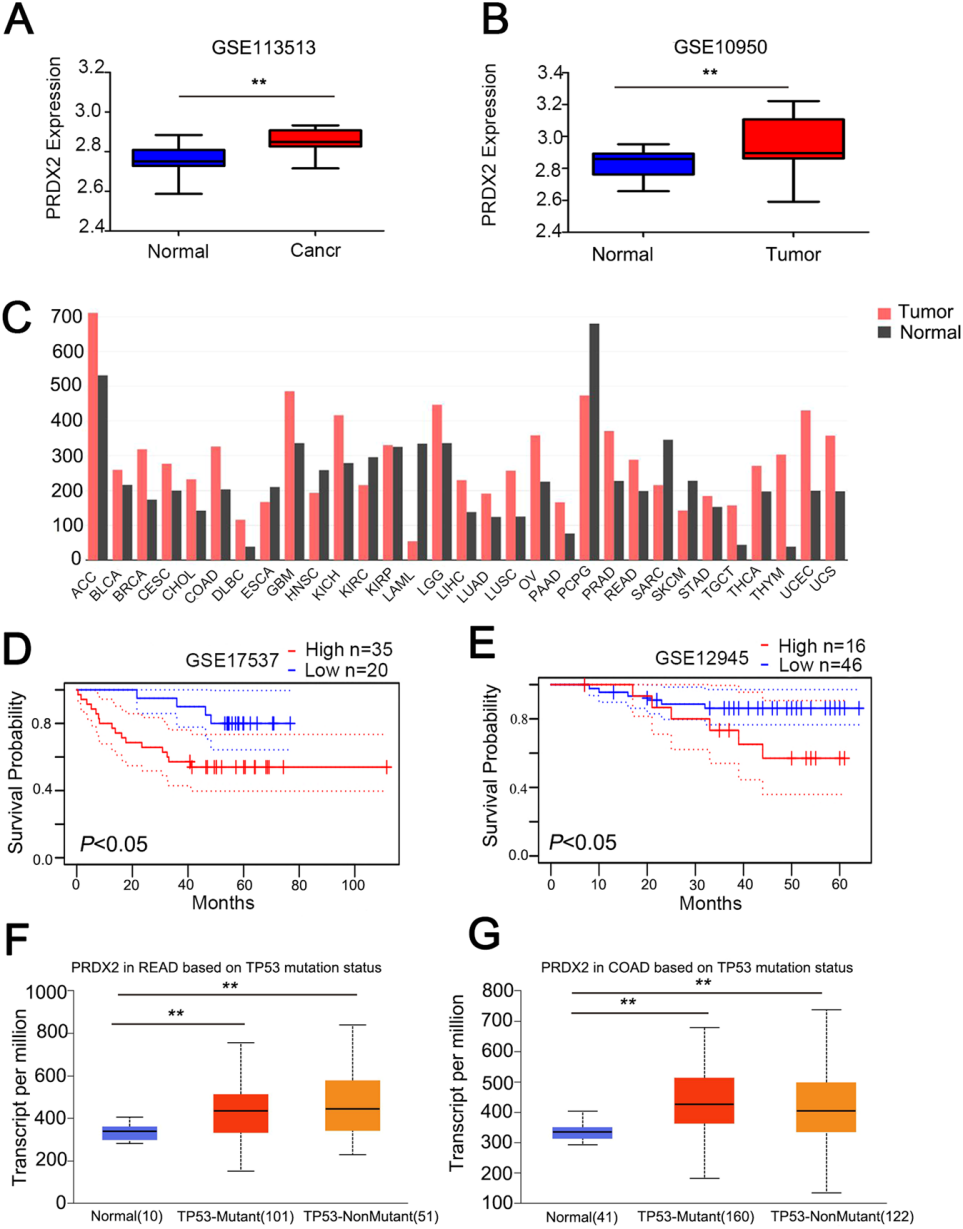
PRDX2 expression in tumor tissues. **A**, **B** PRDX2 expression in the GSE113513 and GSE10950 datasets. **C** PRDX2 mRNA expression in cancers from the GEPIA database. **D**, **E** Kaplan-Meier analysis of PRDX2 expression and survival probability in the GSE17537 and GSE12945 datasets. **F**, **G** PRDX2 expression in p53 mutant and p53 nonmutant samples in the TCGA database.

To determine the mRNA expression of PRDX2 in colorectal cancer, we downloaded the GSE113513 and GSE10950 datasets deposited in the GEO database. In GSE113513 and GSE10950 datasets, bioinformatics analysis revealed that the PRDX2 mRNA level was significantly higher in the cancer tissues compared to the normal tissues (Fig. 1A, B). In the GEPIA database, we also found that PRDX2 expression was higher in most tumors than in adjacent normal tissues (Fig. 1C). In GSSE17537 and GSE12945, Kaplan-Meier analysis showed that patients with low expression of PRDX2 had a longer survival time than those with high expression (Fig. 1D, E). In the TCGA database, we found that PRDX2 expression was higher in READ and COAD tissues than in adjacent normal tissues. The expression of PRDX2 had no relationship with the p53 mutation state (Fig. 1F, G).

### Silencing PRDX2 inhibits colorectal cancer cell proliferation

**Fig. 2:**
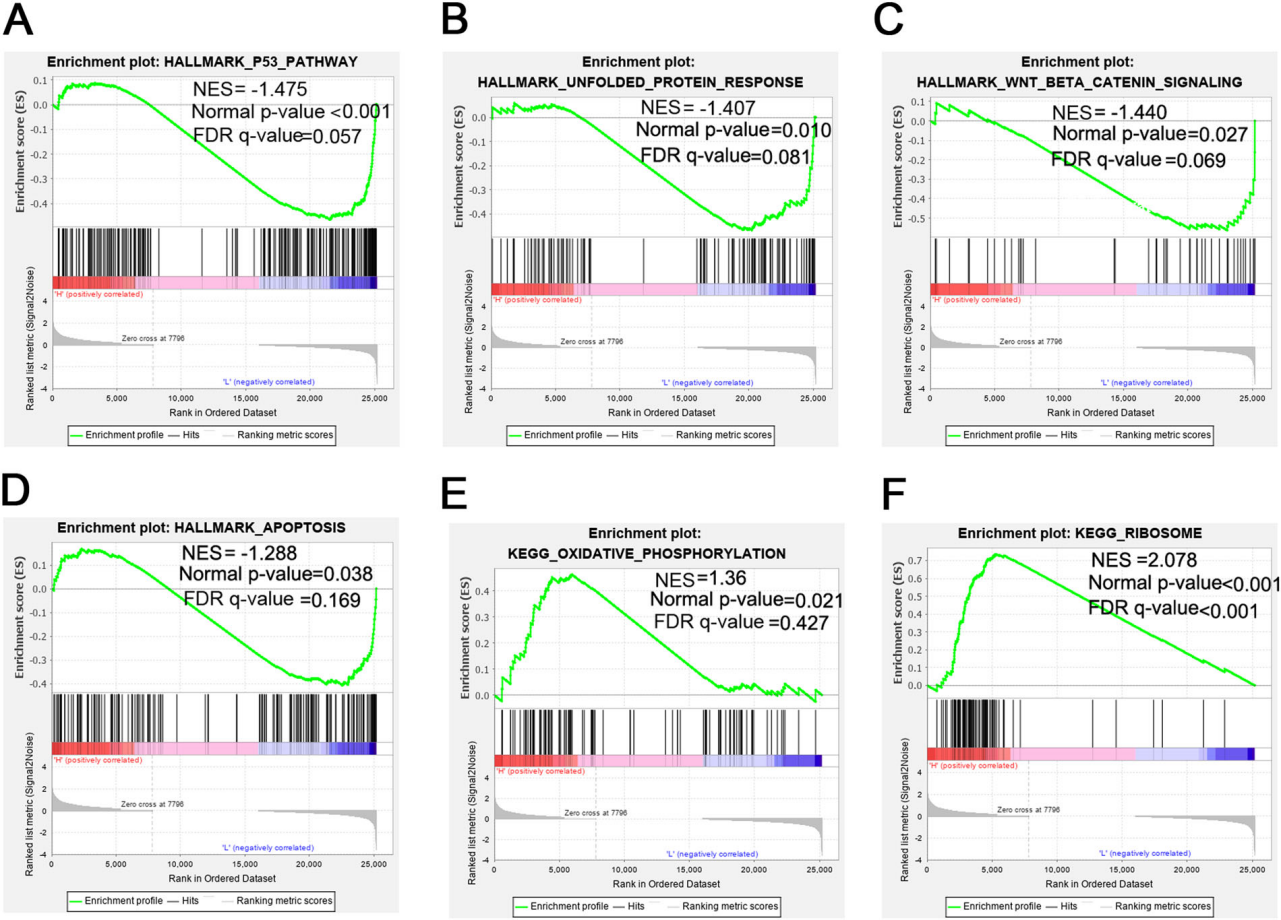
Gene set enrichment analysis based on the microarray dataset GSE81429. Gene sets including: **A** HALMARK_P53_PATHWAY, **B** HALMARK_UNFOLDED_PROTREIN, **C** HALLMARK_WNT_BETA_CATENIN_SIGNALING, and **D** HALLMARK_APOPTOSIS were enriched in the low-PRDX2-expression group. **E** KEGG_OXIDATIVE_PHOSPHORYLATION, and **F** KEGG_RIBOSOME were enriched in the high-PRDX2-expression group. NES, normalized enrichment score; FDR, false discovery rate.

**Fig. 3:**
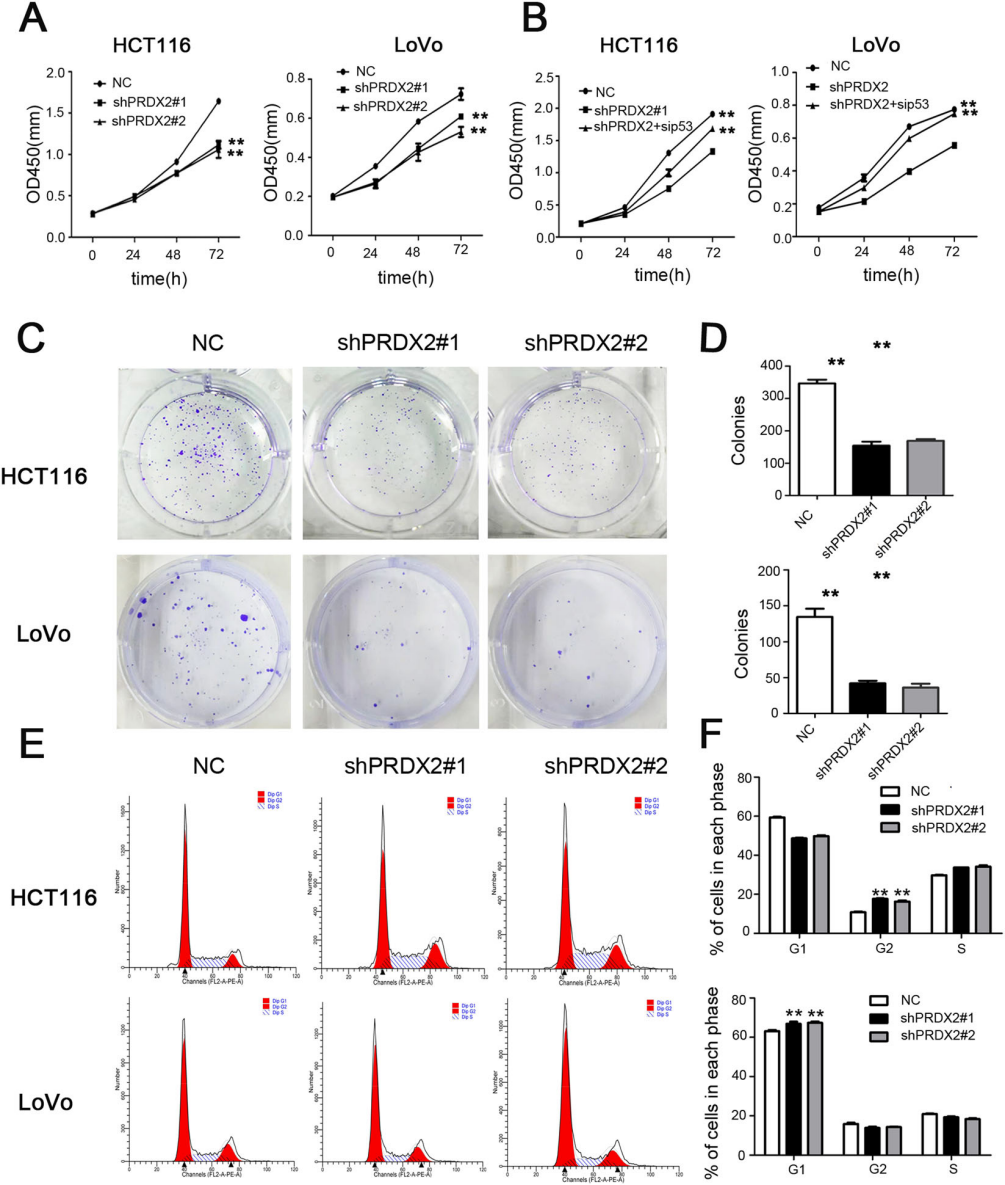
The effect of silencing PRDX2 on the proliferation of HCT116 and LoVo cells. **A** Cell proliferation was assessed by the CCK-8 assay at the indicated times. The data are shown as mean ± SD, *n* = 3, **p < 0.05. **C**, **D** The results of the colony formation assay for the NC and shPRDX2 groups. The data are presented as mean ± SD, *n* = 3, **p < 0.05. **E**, **F** Flow cytometric analysis of cell cycle distribution in the NC and shPRDX2 groups. The percentages of cells in each phase of the cell cycle are shown as mean ± SD, *n* = 3, **p < 0.05.

To study the function of PRDX2 in colorectal cancer, GSEA analysis was applied in the GSE81429 dataset. GSEA analysis revealed that HALMARK_P53_PATHWAY, HALMARK_UNFOLDED_PROTREIN, HALLMARK_WNT_BETA_CATENIN_SIGNALING, and the HALLMARK_APOPTOSIS gene sets were enriched in the low-PRDX2-expression group (Fig. 2A–D). The KEGG_OXIDATIVE_PHOSPHORYLATION and KEGG_RIBOSOME gene sets were enriched in the high-PRDX2-expression group (Fig. 2E, F). To determine the effect of PRDX2 on cell proliferation, we checked cell proliferation with the CCK-8 experiment. Results showed that silencing PRDX2 markedly retarded the proliferation of HCT116 and LoVo cells (Fig. 3A). The colony formation experiment showed that the number of colonies in the shPRDX2 groups was much lower than that in the NC group (*p* < 0.05). An abnormal cell cycle is highly related to cancer mechanisms [22]. To check the effect of PRDX2 on the cell cycle, flow cytometry was used to analyze the changes. The results showed that the cell cycle was arrested in G1 phase in LoVo cells and that the cell cycle was significantly inhibited in G2/M phase in HCT116 cells. The protein p53 is an important cell cycle regulator that could arrest the cell cycle in both G1 and G2/M phases [16]. To determine whether the cell proliferation depends on the function of p53, siRNA targeting p53 was used to treat the cells in the shPRDX2 group. The CCK-8 assay revealed that cell proliferation was restored after the transfection of p53 siRNA, indicating that PRDX2-induced cell proliferation is p53-dependent.

### Silencing PRDX2 induces p53 upregulation

**Fig. 4:**
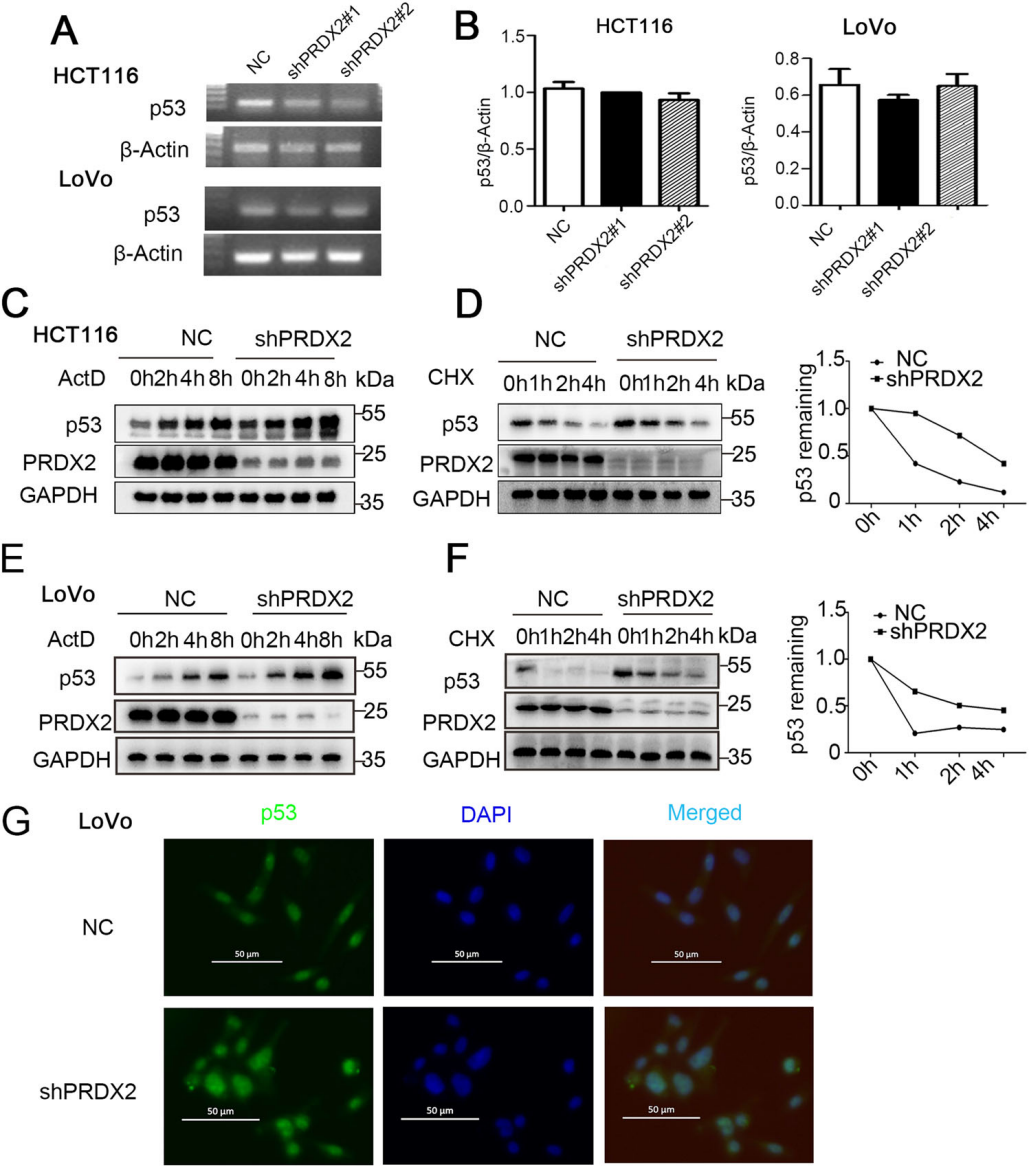
Silencing PRDX2-induced p53 protein upregulation. **A**, **B** RT-PCR was used to measure the mRNA level of p53 in the NC and shPRDX2 groups. **C**, **E** HCT116 and LoVo cells were treated with actinomycin (ActD 5 nM), total protein was harvested at the indicated time point, and p53 protein expression was measured by western blotting. **D**, **F** HCT116 and LoVo cells were treated with cycloheximide (CHX 20 nM). Total protein was harvested at different time points, and p53 protein expression was measured by western blot analysis, quantified by densitometry, and plotted against time to determine the half-life of p53. **G** p53 expression and localization were tested by immunofluorescence. DAPI was used to visualize the nucleus.

To determine the correlation between PRDX2 and p53, we analyzed the two at the mRNA and protein levels. We verified the effect of PRDX2 knockdown on the p53 mRNA level by RT-PCR. We found that the p53 mRNA was not different between the shPRDX2 group and the NC group (Fig. 4A, B). To explore the correlation between PRDX2 and p53 at the protein level, western blotting was used to measure p53 expression. The data showed that the p53 protein in the shPRDX2 group was significantly higher than that in the NC group. Actinomycin (ActD) is an inducer of the nuclear protein response, which can increase the expression of p53. After ActD treatment for 0, 2, 4, and 8 h, whole-cell lysates were collected for western blot analysis. The level of p53 protein in the shPRDX2 group was significantly higher than that in the NC group (Fig. 4C, E). PRDX2 and p53 are negatively correlated at the protein level. To determine whether the half-life of p53 is affected by PRDX2, a cycloheximide (CHX) experiment was employed to verify the changes. After silencing PRDX2, the half-life of p53 extended significantly (Fig. 4D, F). The inhibitory activities of p53 were largely dependent on its nuclear subcellular localization. Hence, immunofluorescence was used to determine whether the upregulated p53 was distributed in the nucleus. Consistently, PRDX2 knockdown increased the fluorescence level of p53, and the upregulated p53 was mainly localized in the nucleus (Fig. 4G). Moreover, nuclear and cytoplasmic proteins were fractionated and further confirmed the nuclear localization of the increased p53 (Supplementary Fig. 1A, B). The above results suggest that silencing PRDX2 does not change the mRNA level of p53 but upregulates the protein level by prolonging the half-life of p53.

### PRDX2 induces the degradation of ubiquitinated p53

**Fig. 5:**
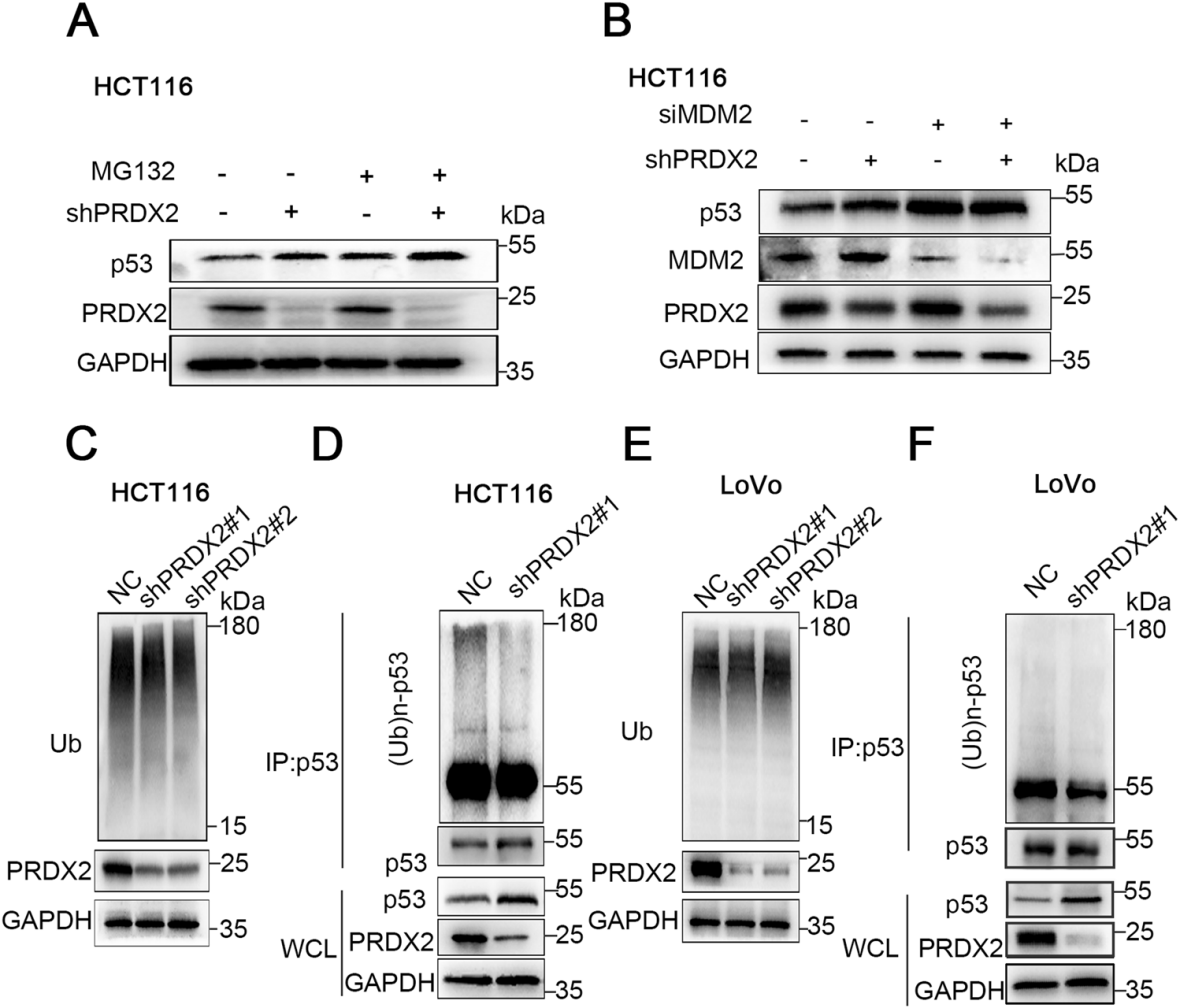
PRDX2 increases MDM2-induced p53 ubiquitination. **A** The degradation of p53 depends on the activation of the proteasome. Cells in the NC and shPRDX2 groups were treated with MG132 (20 µM) for 6 h; p53 and PRDX2 expression was measured by western blotting. **C**, **E** Total ubiquitinated protein levels in the NC and shPRDX2 groups were measured by western blotting. **D**, **F** PRDX2 increased the ubiquitin level of p53. Whole-cell lysates were immunoprecipitated with an anti-p53 antibody; the immunocomplexes were examined by WB with an anti-Ub antibody, and whole-cell lysates were subjected to western blot analysis with the indicated antibodies. **B** p53 ubiquitination was MDM2-dependent. The cells in the NC and shPRDX2 groups were transfected with siMDM2, and the expression of the indicated protein was measured by western blotting.

Proteasomal degradation is an essential process that regulates the protein homeostasis. To confirm whether p53 degradation occurs via proteasomal degradation, MG132 was used to inhibit the proteasome. Results showed that MG132 markedly increased p53 expression (Fig. 5A). p53 is a protein with a short half-life, undergoing rapid ubiquitination and proteasomal degradation. To determine the effect of PRDX2 on total protein ubiquitination and p53 ubiquitination in cells, western blot analysis and immunoprecipitation were used to verify the changes. The results showed that silencing PRDX2 in HCT116 and LoVo cells did not alter the total protein ubiquitination level (Fig. 5C, E). However, immunoprecipitation experiments showed that the ubiquitination of p53 was markedly reduced after PRDX2 knockdown (Fig. 5D, F). Many E3 ligases are involved in the degradation of p53 ubiquitin, among which MDM2 is the most critical one. To determine whether the degradation of p53 depends on MDM2, siRNA was used to knockdown MDM2. Results showed that p53 protein was significantly increased after siMDM2 transfection, indicating that PRDX2-induced p53 ubiquitination is MDM2-dependent (Fig. 5B).

### PRDX2 disrupts the MDM2-RPL4 interaction by binding to RPL4

**Fig. 6:**
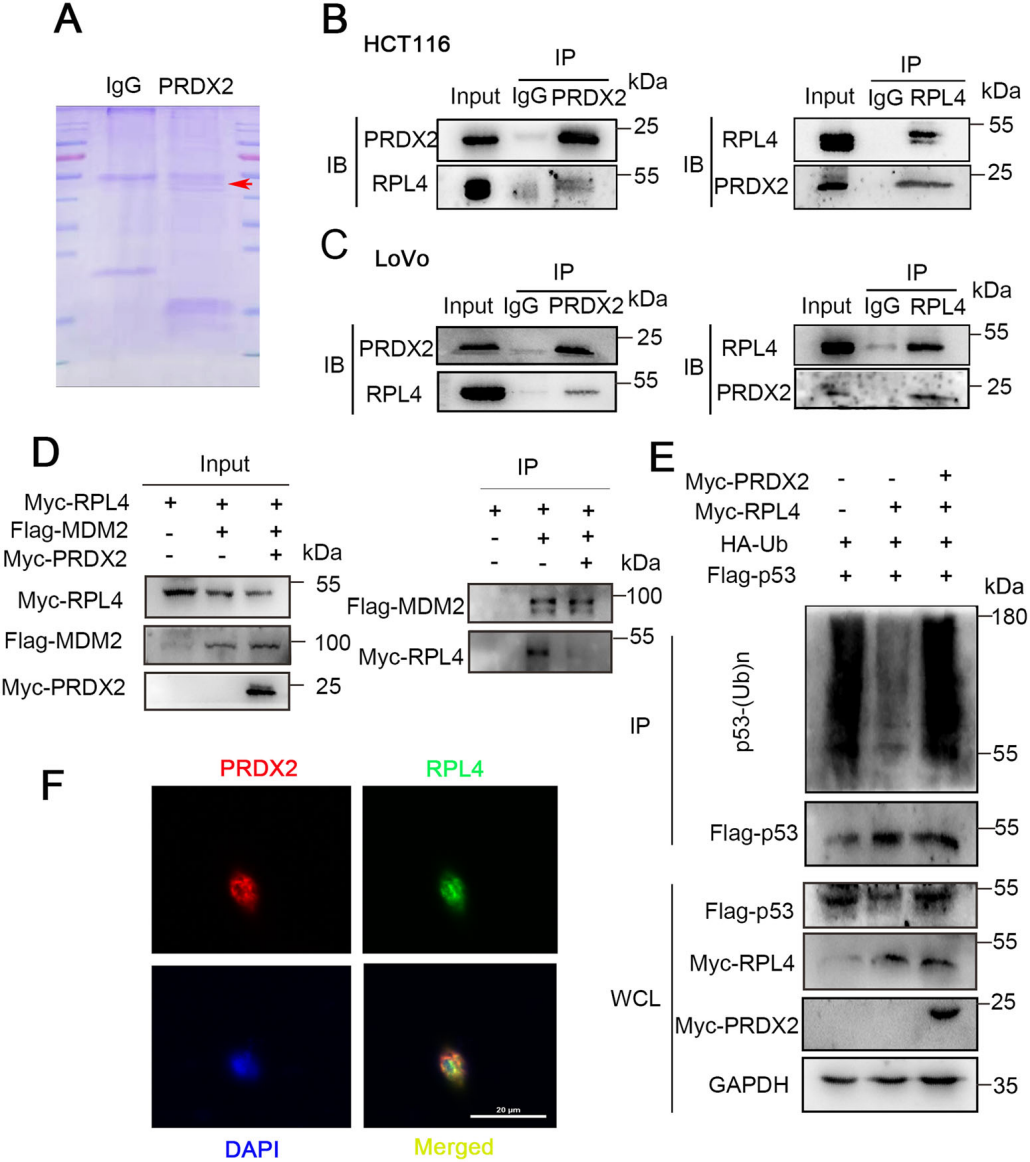
PRDX2 disrupts the RPL4-MDM2 interaction. **A** Endogenous PRDX2 was immunoprecipitated with an anti-PRDX2 antibody, and IgG was used as the negative control. The immunocomplexes were analyzed by western blotting, and then the SDS-PAGE gel was stained with Coomassie brilliant blue. **B**, **C** Endogenous PRDX2 and RPL4 were immunoprecipitated with anti-PRDX2 and anti-RPL4 antibodies, and IgG was used as the negative control. The immunocomplexes were analyzed by western blotting. **D** PRDX2 disrupts the RPL4 and MDM2 interaction. HEK293T cells were cotransfected with pCMV-MDM2-Flag, pCMV-RPL4-Myc, and pCMV-PRDX2-Myc. MDM2-Flag was pulled down by anti-Flag beads. MDM2-Flag and RPL4-Myc were examined with anti-Myc and anti-Flag antibodies. **E** PRDX2 enhances MDM2-mediated p53 proteasomal degradation. HEK293T cells were cotransfected with pCMV-p53-Flag, pCMV-Ub-HA, pCMV-RPL4-Myc, and pCMV-PRDX2-Myc plasmids and treated with MG132 for 6 h before being harvested for an in vivo ubiquitination assay. Input proteins were examined by WB analysis with the indicated antibodies, and Flag-p53 was pulled down by anti-Flag beads and assessed with anti-HA antibodies. **F** Immunofluorescence was used to examine the colocalization of RPL4 and PRDX2 in LoVo cells.

PRDX2 is an important ROS scavenger. To find whether ROS affects the expression of PRDX2, H_2_O_2_ was utilized to generate oxidative stress. Results showed that H_2_O_2_ did not change the PRDX2 expression (Supplementary Fig. 1C, D). To investigate whether p53 upregulation was affected by ROS, *N*-acetyl-cysteine (NAC) was used to scavenge the intracellular ROS. Results showed that NAC did not abolish the p53 upregulation caused by PRDX2 knockdown. These data indicated that p53 upregulation is ROS-independent (Supplementary Fig. 1E, F). Therefore, there must be other mechanisms involved in the p53 regulation. Previous GSEA analysis revealed that the KEGG_RIBOSOME gene set was significantly enriched in the high-PRDX2-expression group (Fig. 2F). Many studies reported the relationship between RPLs-MDM2 and p53. Is there a relationship between PRDX2 and ribosomal proteins? To ascertain our hypothesis, we performed immunoprecipitation and mass spectrometry. The results showed that RPL4 could bind PRDX2. To confirm the results, we performed coimmunoprecipitation and GST-pulldown experiments. We found that RPL4 could bind PRDX2 in HCT116 and LoVo cells (Fig. 6A–C and Supplementary Fig. 1G). Immunofluorescence also revealed the colocalization of PRDX2 and RPL4 in the nucleus and cytoplasm (Fig. 6F). To confirm whether PRDX2 disrupts the binding of RPL4 and MDM2, we transfected pCMV-MDM2-Flag, pCMV-RPL4-Myc, and pCMV-PRDX2-Myc into HEK293T cells. The results showed that RPL4 could bind MDM2; however, after transfection with PRDX2, the binding of RPL4 and PRDX2 decreased (Fig. 6D). We transfected pCMV-p53-Flag, pCMV-Ub-HA, pCMV-RPL4-Myc, and pCMV-PRDX2-Myc into HEK293T cells to determine the mechanism. The results showed that the ubiquitination of p53 was significantly reduced after transfection with RPL4, and PRDX2 restored the p53 ubiquitination (Fig. 6E). These results suggest that PRDX2 may affect the MDM2-RPL4-p53 pathway by binding to RPL4, leading to increased p53 ubiquitination by MDM2.

### Silencing PRDX2 inhibits xenograft tumor growth

**Fig. 7:**
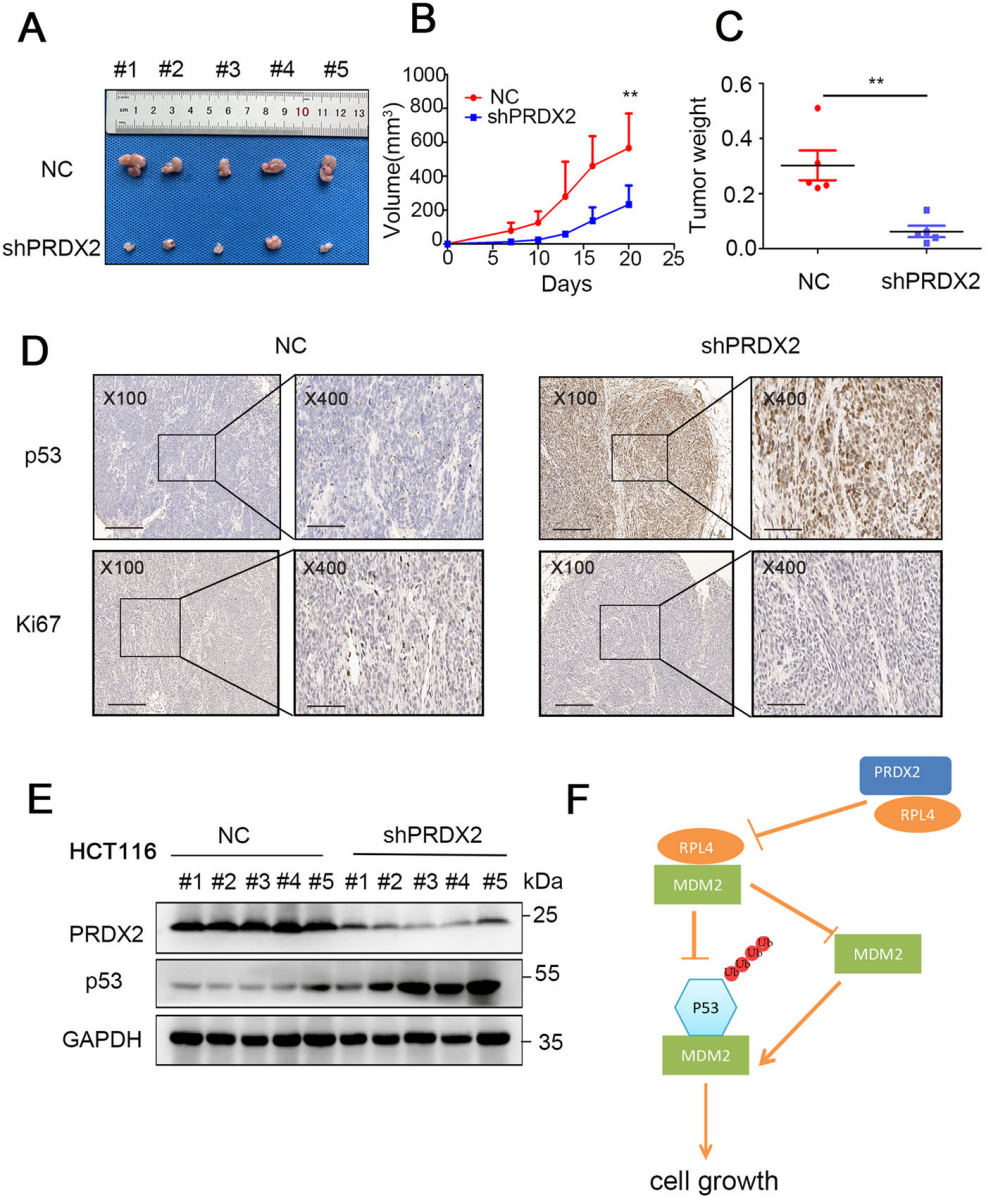
PRDX2 knockdown retards tumor growth by inducing p53 upregulation. **A** Images of xenograft tumors harvested at the end of the experiment. **p < 0.05, by two-tailed *t*-test. **B** Growth curve of tumor in the NC group and the shPRDX2 group in nude mice. **C** Tumor weight of the NC group and the shPRDX2 group in nude mice. **D** p53 and Ki67 were detected in tumors by ICH. **E** The protein levels of PRDX2 and p53 in 5 tumor samples were measured by western blotting with the indicated primary antibodies. **F** the working model summarizes the research.

To investigate whether PRDX2 plays an oncogenic role in vivo, we performed a xenograft tumor growth experiment by subcutaneously injecting shPRDX2-HCT116 and NC-HCT116 cells into the dorsal flank. The results showed the growth rate in the NC group was higher than that in the shPRDX2 group, and that the tumor volume in the NC group was markedly larger than that in the NC group (Fig. 7A–C). To further confirm the previous results, western blotting was used to check p53 and PRDX2 expression in xenograft tumor tissue. We found that shPRDX2 significantly increased p53 protein abundance (Fig. 6E). Accordingly, immunohistochemistry revealed that silencing PRDX2 reduces the proliferation marker Ki67 (Fig. 6D).

## Discussion

Our previous study showed that PRDX2 is closely correlated with poor differentiation, advanced stage, lymph node metastasis of colorectal cancer [8]. The prognosis of patients with higher PRDX2 expression is worse than that of patients with low expression [8]. These results suggest that PRDX2 plays an oncogenic role in colorectal cancer. In line with previous findings, we found that PRDX2 expression was upregulated in the GSE113513 and GSE10950 datasets. PRDX2 knockdown inhibited cancer cell growth, colony formation, and xenograft tumorigenesis derived from human colorectal cancer cells. Kang et al. reported that PRDX2 did not inhibit cell proliferation in RKO cells, which is not consistent with the above findings [23]. All these results indicate that PRDX2 plays a very complicated role in the occurrence and development of tumors. As a well-known tumor suppressor, p53 plays a vital role in cell cycle transformation [24]. When p53 is activated, the expression of many cycle-related proteins is downregulated, such as cyclin B1, cyclin A, CDK1, cyclin B2, and CDC20. The reduction in cycle-related protein expression can inhibit cell cycle transformation and induce tumor cell apoptosis [25–28]. p53 is one of the most commonly mutated genes, and p53 mutations can be found in 50% of colorectal cancer samples [29]. Both missense p53 mutations and loss of wild-type p53 can promote the occurrence and development of tumors. We found that silencing PRDX2 can significantly increase p53 protein expression. Besides, PRDX2 is reported to be upregulated in many other tumors, such as gastric cancer, and lung cancer, suggesting that PRDX2 may be a promising prognostic biomarker [8, 10].

Proteins are usually regulated by posttranslational modifications, of which ubiquitylation is the second most common only behind phosphorylation [30]. Protein ubiquitination dysregulation is one of the causes of colorectal cancer. von Hippel Lindau (VHL) gene mutations have been found in 10% of colorectal cancer [31]. The VHL protein is a component of a protein complex that contains Elongin B/C, Cul2, and RBX1. This complex functions as an E3 ubiquitin ligase which regulates the degradation of HIF1α [32, 33]. Mutations of VHL can cause HIF1α stabilization, inducing sustained tumor angiogenesis [31]. APC mutation stabilizes β-catenin which in turn leads to the activation of the Wnt signal pathway [34]. p53 is regulated by intricate posttranslational modifications, such as phosphorylation, ubiquitination, methylation, and glycosylation [35]. MDM2 ubiquitinates p53 via lysine-48-based polyubiquitination and is ultimately degraded by the 26S proteasome [36–38]. Single or lysine-63-based polyubiquitination can lead to p53 nuclear export and cytoplasmic relocation, ultimately leading to stabilization of p53 [39, 40]. In our study, CHX experiments revealed that the half-life of p53 increased in the shPRDX2 group. MG132 restored PRDX2-induced p53 degradation. Immunoprecipitation experiments showed PRDX2 increased p53 ubiquitination. These results suggested that PRDX2-induced colorectal cancer proliferation has a close relation with p53 ubiquitination dysregulation.

After silencing MDM2 with siRNA, the degradation of p53 was restored, suggesting that PRDX2-induced p53 reduction is MDM2-dependent. The RPL-MDM2-p53 pathway has been reported to regulate the expression of p53 [41–44]. The ribosomal protein response is usually activated under stress [45]. Ribosomal protein could bind to MDM2 [46, 47], which prevents the interaction between MDM2 and p53, causing decreased p53 ubiquitination and degradation [48]. Whether this pathway is regulated by other proteins remains largely elusive. Previous immunoprecipitation and mass spectrometry experiments revealed that PRDX2 can bind RPL4 and many other proteins. The results of coimmunoprecipitation and GST-pulldown once again confirmed that PRDX2 and RPL4 exhibit specific binding in HCT116 and LoVo cells. Furthermore, we found that PRDX2 and RPL4 were colocalized in the nucleolus by immunofluorescence. The interaction between PRDX2 and RPL4 inhibited the binding of RPL4 and MDM2, relieved the functional inhibition of RPL4 on MDM2, led to the ubiquitination of p53, and promoted protein degradation. External stimuli such as chemotherapy and radiotherapy can activate the ribosome response and increase p53-dependent cell cycle arrest and apoptosis [45]. However, in tumor cells with high PRDX2 expression, PRDX2 could bind to RPL4, resulting in decreased p53 levels. This may be a potential mechanism of PRDX2-dependent tumor proliferation or drug resistance [49]. Proteins are the real mediators of cellular activities; therefore, targeting proteins is a preferred choice for cancer-targeted therapy. Interfering with the interaction between PRDX2 and RPL4 may reverse the chemoresistance of tumor cells.

In summary, we found that PRDX2 is highly expressed in colorectal cancer tissues and can promote colorectal cancer cell proliferation. More importantly, we found that PRDX2 can bind RPL4, hindering the RPL4-MDM2 binding, and leading to p53 degradation (Fig. 7F), thereby promoting tumor proliferation. Strategies targeting PRDX2 may be developed as therapies for colorectal cancer.

## Materials and methods

### Cell culture and antibodies

HCT116, LoVo, and human HEK293T cells were purchased from the Shanghai Cell Bank of Type Culture Collection (Shanghai, China). HEK293T and HCT116 cells were maintained in Dulbecco’s modified Eagle’s medium (Gibco, NY, USA) supplemented with 15% fetal bovine serum (Gibco, NY, USA). LoVo cells were maintained in F12/DMEM (Gibco, NY, USA) supplemented with 15% fetal bovine serum. All the cells were cultured at 37 °C in a 5% CO_2_ humidified atmosphere. The sources of antibodies against the following proteins were as follows: PRDX2 (10545-2-AP,60202-1-Ig), RPL4 (11302-1-AP), P53 (10442-1-AP), MDM2 (19058-1-AP), GAPDH (10494-1-AP), Ubiquitin (10201-2-AP), ki67 (27309-1-AP), Flag (66008-3-Ig, 20543-1-AP), Myc (16286-1-AP, 60003-2-Ig) from Proteintech Group; HA (#3724), GST (#2624), PCNA (#13110S) from Cell Signaling Technology.

### Cell viability assay

HCT116 cells and LoVo cell suspensions were seeded at a density of 3000 cells per well in 96-well plates. According to the manufacturer’s instructions, cell viability was measured every 24 h for 3 days using the Cell Counting Kit (Dojindo Laboratories, Kumamoto, Japan). Each sample’s absorbance value was measured at 450 nm with a microplate reader (Infinite 2000 PRO, TECAN, Männedorf, Switzerland).

### Colony formation assay

HCT116 cells and LoVo cells were trypsinized and seeded in a 6-well plate at a density of 500 cells per well and cultured until colonies were visible. The cells were fixed with 4% paraformaldehyde and stained with crystal violet blue solution (Beyotime, C0121) at room temperature for 30 min.

### Cell cycle analysis

HCT116 cells and LoVo cells were trypsinized and fixed in 75% ice-cold ethanol for 24 h at 4 °C. The cells were washed with PBS twice and then incubated with 100 µl RNase A (0.1 mg/ml) at 37 °C for 20 min. Then, 2 µl propidium iodide was added to the suspension and incubated at 37 °C for another 20 min. The cell cycle was analyzed using a FACS Calibur flow cytometer (BD Biosciences, Franklin Lakes, NJ, US).

### siRNA, plasmids transfection, and lentivirus infection

siRNAs against p53 and MDM2 and a negative control siRNA were purchased from GenePharma (Shanghai, China). siRNAs (40 nM) were transfected using Lipofectamine 2000 (Invitrogen, Carlsbad, CA, USA) following the manufacturer’s instructions. The sequences of the siRNAs targeting p53 and MDM2 were GACTCCAGTGGTAATCTAC and AGGCAAATGTGCAATACCA, respectively. The expression vectors pCMV-p53-Flag, pCMV-PRDX2-Myc, pCMV-RPL4-Myc, pCMV-HA-Ub, and pCMV-MDM2-Flag were constructed in our laboratory. Lentiviruses silencing PRDX2 were constructed by GenePharma. HCT116 cells and LoVo cells were infected with lentiviruses according to the manufacturer’s instructions. Puromycin (2–6 µg/ml) was used for stable cell line selection. The sequences of the specific shRNA targeting PRDX2 were GTGAAGCTGTCGGACTACAAA (shPRDX2#1) and CCTTCGCCAGATCACTGTTAA (shPRDX2#2).

### Immunohistochemistry (IHC)

Slides were deparaffinized in xylene, then, heat-mediated antigen retrieval was performed using citrate buffer. The samples were incubated with primary antibodies against p53 or Ki67 at 4 °C overnight and then with secondary antibody at room temperature for 30 min. All immunostained images were obtained using a microscope.

### Western blot analysis

Equal amounts of 20 µg of total protein were separated by 10% sodium dodecyl sulfate-polyacrylamide gel electrophoresis, and then the proteins were transferred onto PVDF membranes (Bio-Rad, Hercules, CA, USA). All PVDF membranes were blocked in skim milk for 1 h at room temperature and then incubated with primary antibodies at 4 °C overnight. The next day, the membranes were washed with TBST and incubated with an HRP-conjugated secondary antibody at room temperature for 1 h. An ECL chemiluminescence system was used to visualize the immunoreactive bands.

### RT-PCR

Total RNA was extracted from the cells using TRIzol reagent (Invitrogen, CA, USA). RT-PCR was performed with Go-Taq (Promega, WI, USA), and β-Actin was amplified as an internal control. The sequences of the β-Actin primers were F: TCCTGTGGCATCCACGAAACT and R: GAAGCATTTGCGGTGGACGAT. The sequences of the p53 primers were F: ACCTATGGAAACTACTTCCTGAAA, R: CTGGCATTCTGGGAGCTTCA.

### In vivo ubiquitination assay

The indicated plasmids were transfected into HEK293T cells. Before harvesting the protein, HEK293T cells were treated with 40 μM MG132 (Selleck, S2619) for 6 h. At 48 h after transfection, the cell lysate was incubated with anti-Flag beads (Bimake, B26101) overnight at 4 °C. The next day, the anti-Flag beads were washed three times with 0.5% PBST. The immunocomplexes were subjected to western blot analysis with the indicated antibodies.

### Coimmunoprecipitation

Cells were lysed with NP40 (Beyotime, P0013F), and 1000 mg protein was incubated with the indicated antibodies at 4 °C for 4 h. Fifty microliters of Protein A/G Agarose beads (Beyotime, P2006) were then added to the samples, and the mixture was incubated at 4 °C for 2 h on a rocking platform. The beads were collected and washed three times with lysis buffer. The immunocomplexes were subjected to western blot analysis with the indicated antibodies.

### Immunofluorescence staining

HCT116 and LoVo cells were trypsinized and seeded on glass coverslips. The cells were fixed in 4% paraformaldehyde for 10 min followed by 0.5% Triton X-100 for 10 min. After blocking with 5% bovine serum albumin, the fixed cells were incubated with the indicated primary antibodies at 4 °C overnight. The next day, the cells were washed with PBS solution three times and then incubated with secondary antibodies and 4',6-diamidino-2-phenylindole (Beyotime, C1002) for nuclear staining.

### Bioinformatics analysis

GSEA version 4.0.3 (Broad Institute, USA) was used to analyze the GEO datasets. The gene sets “h.all.v7.2.symbols.gmt” and “c2.cp.kegg.v7.2.symbols.gmt” were downloaded from the Molecular Signatures Database (http://software.broadinstitute.org/gsea/msigdb/index.jsp) and used for the enrichment analysis. We selected 1000 permutations to calculate the normalized enrichment score (NES), normal *p*-value, and false discovery rate (FDR *q*-value).

### GST-pulldown assay

The GST-PRDX2 fusion protein was expressed in BL21-DE3 *Escherichia* *coli* and was incubated with Glutathione-Sepharose beads (Beyotime, P2262) at 4 °C for 1 h to generate GST conjugates. HCT116 cell lysates were then incubated with the GST-conjugated beads at 4 °C overnight. The complexes were washed with lysis buffer four times, analyzed with western blot.

### Nude mouse xenograft experiments

Six-week-old female nude mice were purchased and randomly allocated into NC and shPRDX2 group (five in each group). Xenografts were generated by subcutaneously injecting 5 × 10^6^ HCT116-shPRDX2 and control cells into the dorsal flank. The tumors were observed and measured every 2 days. Tumor volumes were calculated by the following formula: ½ (*L* × *W*^2^).

### Statistical analysis

Statistical differences in our data were analyzed using IBM SPSS 22.0 software. All in vitro experiments were independently performed at least three times. Student’s *t*-test or one‐way analysis of variance (ANOVA) was used to analyze the differences between groups. All data are presented as mean ± SD. A *p*-value < 0.05 was considered statistically significant.

## Supplementary information

Supplementary Fig. 1.

Supplementary Fig. 2.

Supplementary Figure Legends.

IP-MS.
