# Supplementary figures and images for: PRDX2 promotes the proliferation of colorectal cancer cells by increasing the ubiquitinated degradation of p53
Source: Cell Death Dis. 2021 Jun 11;12(6):605. doi: 10.1038/s41419-021-03888-1 (PMC8196203; doi:10.1038/s41419-021-03888-1)

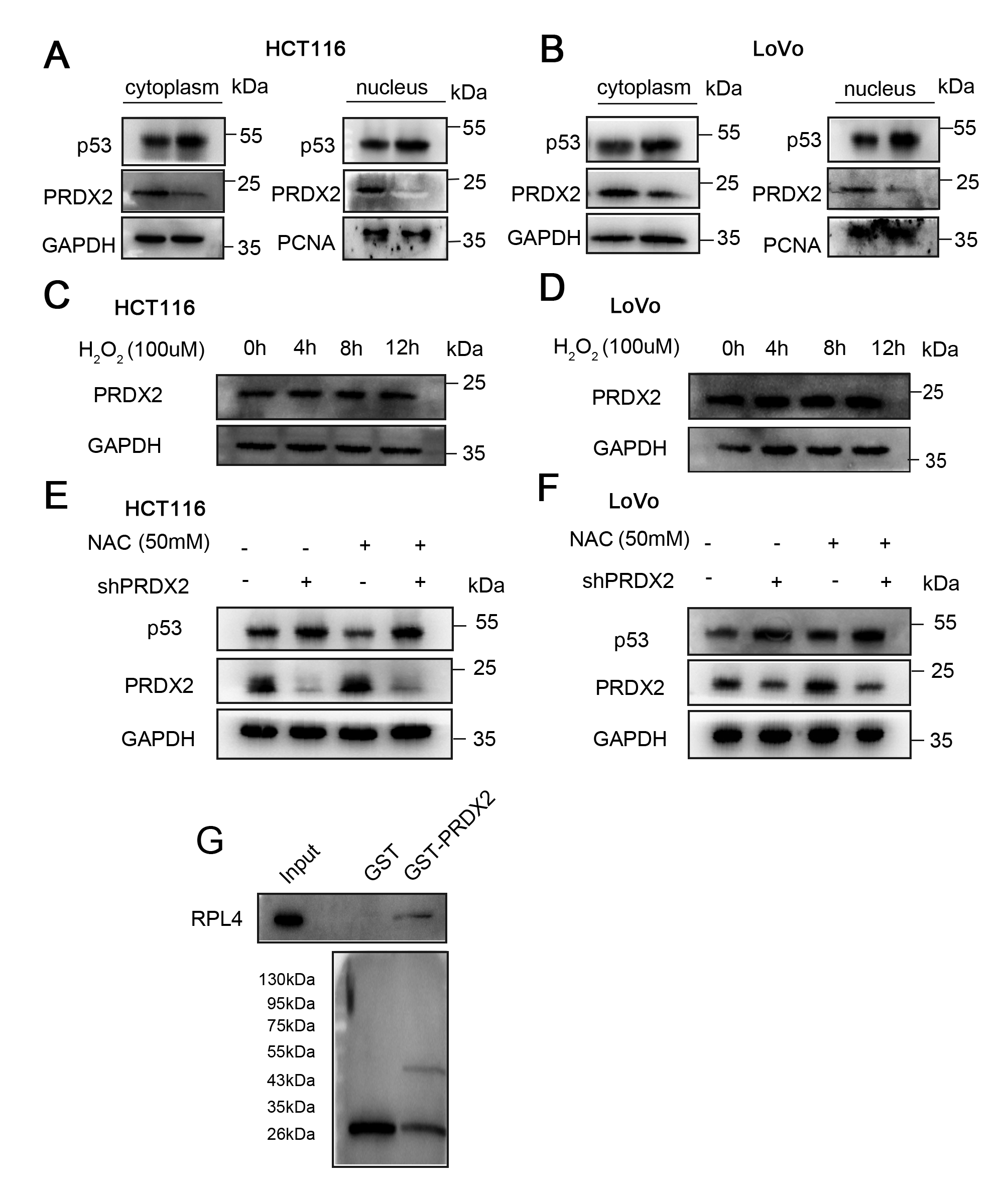

Supplement: Supplementary file 1 — Supplementary Fig. 1. [file 41419_2021_3888_MOESM1_ESM.tif]

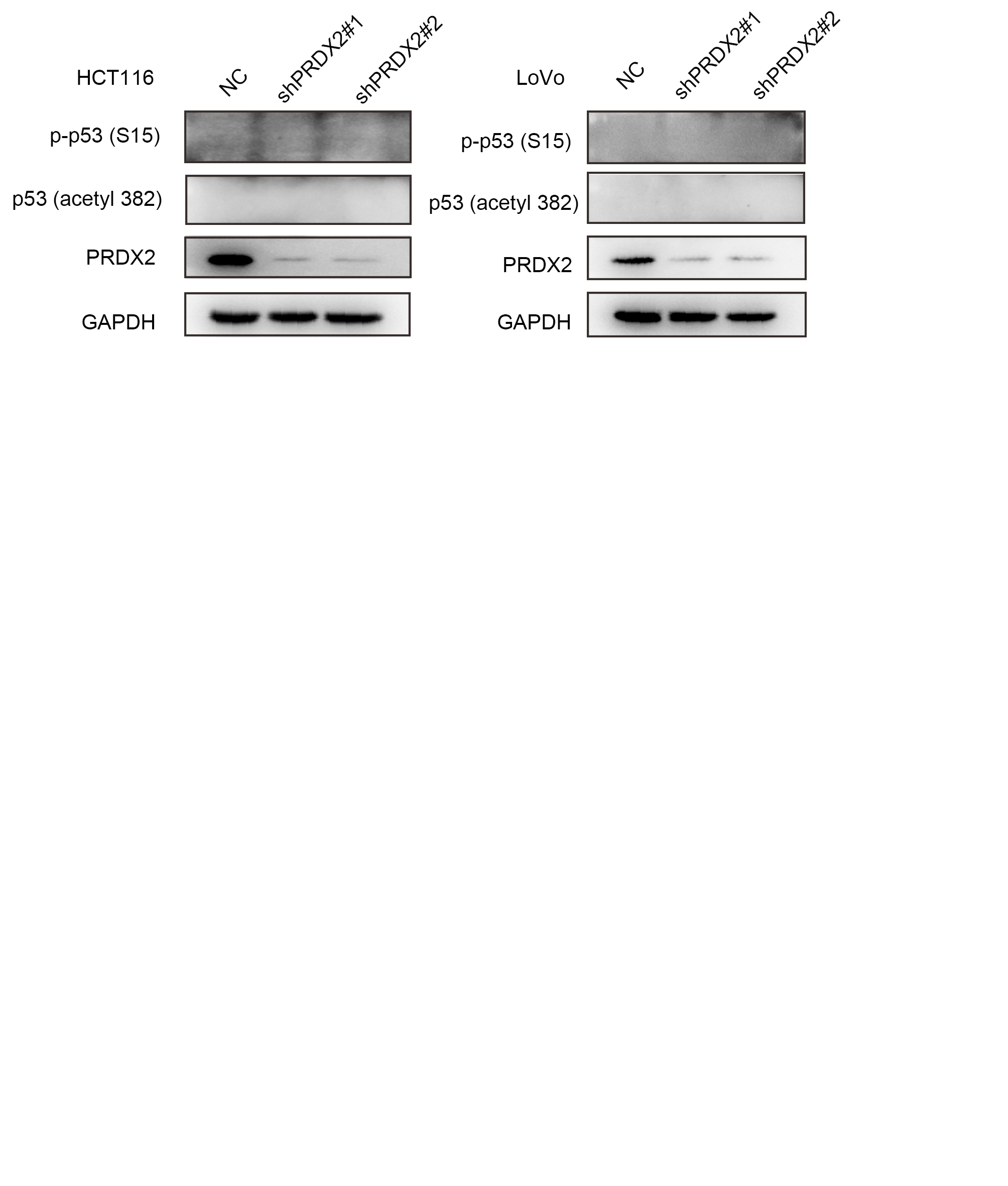

Supplement: Supplementary file 2 — Supplementary Fig. 2. [file 41419_2021_3888_MOESM2_ESM.tif]
