# Supplementary material for: PRDX2 promotes the proliferation of colorectal cancer cells by increasing the ubiquitinated degradation of p53
Source: Cell Death Dis. 2021 Jun 11;12(6):605. doi: 10.1038/s41419-021-03888-1 (PMC8196203; doi:10.1038/s41419-021-03888-1)
Supplement: Supplementary file 3 — Supplementary Figure Legends. [file 41419_2021_3888_MOESM3_ESM.docx]

**Supplementary Figure 1.** (A, B) Silencing PRDX2 induced p53 protein upregulation and nuclear subcellular localization. Nuclear and cytoplasmic cell lysates were prepared from HCT116 and LoVo cells. Changes of p53 and PRDX2 were examined by western blotting. (C, D) ROS does not affect the expression of PRDX2. H_2_O_2_ (100uM) was utilized to generate oxidative stress and PRDX2 was tested by western blotting. (E, F) Silencing PRDX2 induced p53 upregulation is independent of ROS. NC and shPRDX2 groups were treated with NAC (50mM) for 12 hours. p53 and PRDX2 were measured by western blotting. (G) GST and GST-PRDX2 fusion protein was immobilized on glutathione-sepharose beads and incubated with HCT116 cell lysates at 4℃overnight. Purified GST, GST-PRDX2, and RPL4 were detected with indicated antibodies.

**Supplementary Figure 2.** p53 phosphorylation and p53 acetylation were detected with indicated antibodies.
